# Zinc bioavailability in semiarid agricultural regions: a soil property-based assessment

**DOI:** 10.1007/s10653-025-02544-3

**Published:** 2025-05-31

**Authors:** Kadriye Taşpınar, Halil Aytop, Özgür Ateş, Memet Varol, Gülser Yalçın, Fatih Kızılaslan, Hasan Çakıllı, Serdar Toprak

**Affiliations:** 1 Soil and Water Research Department, Transitional Zone Agricultural Research Institute, Eskisehir, Türkiye; 2 Soil and Water Research Department, East Mediterranean Transitional Zone Agricultural Research of Institute, Kahramanmaraş, Türkiye; 3 Aquaculture Enginnering Department, Agriculture Faculty, Malatya Turgut Özal University, Malatya, Türkiye; 4 Plant Production Department, Söke Directorate of Agricultural Production Enterprise, Agricultural Extension and In-Service Training Center, Aydın, Türkiye

**Keywords:** Zn accumulation, Available Zn deficiency, Soil property, Spatial variation of available and total Zn, Semiarid region

## Abstract

Zinc concentration in soils can cause both nutritional deficiency and toxicity in plants. Therefore, both the concentration and availability of Zn, especially in semiarid soils, are critical for agriculture and the environment. This study was carried out to determine the relationship between Zn concentrations and some soil properties in semiarid agricultural areas of Türkiye and to create spatial distribution maps. For this purpose, 1529 surface soil samples (0–30 cm) were taken from the 8687 km^2^ study area and organic matter (OM), calcium carbonate (CaCO_3_), pH, available phosphorus (AP), available Zn (AZn) and total Zn (TZn) analyses were carried out. In addition, the Zn availability ratio (Zn-AR) was calculated by the ratio of AZn to TZn concentration. The results indicated that AZn and TZn had high coefficients of variation (> 45%) due to the high heterogeneity of agricultural soils in the study area. Total Zn concentration ranged from 11.74 to 276.45 mg kg^−1^ and only 11.8% of the soil samples for TZn were found to exceed the upper continental crust value (67 mg kg^−1^), indicating low Zn accumulation in the study area. Similarly, none of the samples for TZn exceeded the maximum permissible concentration in soils. However, AZn deficiency was determined in 55% of the samples. Correlation analysis showed that AZn had significant positive correlations with TZn, AP and OM (*p* < 0.01). Spatial distribution maps showed that there were some differences in distribution trends of TZn and AZn concentrations. Total Zn concentrations were higher in the northern and northwestern regions of the study area, while AZn concentrations were higher in the western region. The zinc availability ratio (Zn-AR) showed higher values in the western and southeastern regions of the study area. Soil properties influencing the spatial distribution of Zn availability were AZn, AP and OM.

## Introduction

Zinc (Zn) is one of the microelements that plays a role in plant growth and development and in the protection of human and animal health. Approximately one-third of the population in developing countries, especially women and children, are significantly affected by Zn deficiency (Ghandilyan et al., 2006; Cakmak 2017; He et al., 2021; Suganya et al., 2020). In addition, Zn is one of the 17 elements required for plant development and takes part in basic developmental processes such as gene expression, photosynthesis, pollen development, protein synthesis, signal transduction, cofactor of enzymes, and auxin mechanism (Hacisalihoglu, 2020). It has also been reported that Zn may accumulate in soil and water and cause contamination (Cakmak, 2008; Noulas et al., 2018). Zinc deficiency in humans occurs when the products grown in Zn-deficient soils are consumed in the diet (Alloway, 2009). Zn concentrations in unfertilized, unpolluted, natural soils vary from 10 to 300 mg kg^−1^. The Zn concentration of such soils is closely related to the chemical composition and weathering processes of the bedrock ( Noulas et al., 2018). The lowest Zn values are found in sandy and high calcareous soils, while some researchers cite the average Zn concentration as 70 mg kg^−1^ for soils worldwide (Kabata-Pendias, 2010). Zn in the soil is in the soil solution and binds to clay minerals, carbonates or organic matter (Recena et al., 2021). Since Zn has a strong affinity for colloidal particles in the soil, the utilization of Zn by plants decreases (Duffner et al., 2014). According to estimates, 50% of the world’s cereal production areas are assumed to be Zn deficient (Cakmak & Kutman, 2018). Zinc deficiency in agricultural soils limits plant growth by disrupting many physiological and biochemical processes and may cause yield losses exceeding 40% in product yield (Noulas et al., 2018; Zeng et al., 2021).

Soil degradation, erosion, a loss of organic matter, salinization, and pollution are the most serious threats to the soil (European Commisssion 2006; Aytop & Şenol, 2022a). Using and managing lands according to their abilities is one of the most basic ways to minimize these threats to the soil (Aytop & Şenol, 2022b). Therefore, proper soil nutrient management is critical for meeting the needs of the world’s ever-increasing population while preserving the environment, soil and human health (Wu et al., 2010). Knowing the ratio of available Zn concentration to total Zn concentration in agricultural soils and used fertilizers is important for the characterization of soils. Soil studies and maps showing the geographical distribution of the concentrations of microelements in soils that are important for plant nutrition will guide for the sustainable management of nutrients in the soil. The type and extent of micronutrient deficiencies and toxicities in plants, animals, and people can be better understood using such inventory data (Wu et al., 2010).

Although studies on Zn have been conducted in recent years, most of these studies have focused on eliminating Zn deficiency in products grown in regions where Zn deficiency is observed (Candan et al., 2018; Imran et al., 2015; Nikolic et al., 2016; Obaid et al., 2022; Phuphong et al., 2020; Watts-Williams et al., 2017; Zeng et al., 2021; Zhao et al., 2020). Few studies have been conducted to determine and characterize the variability of Zn concentration in agricultural soils concerning some soil properties. Therefore, in order to prevent nutrient deficiencies and soil pollution, especially in semiarid regions, the plant nutrient concentration of soils should be examined in detail (Moreno-Jiménez et al. 2022). In semiarid regions, agricultural crop diversity and productivity are more limited compared to rainy regions. Cultivation practices should be optimized to sustain food production in these regions. Considering that a significant part of Türkiye has a semiarid climate, determining soil factors that limit plant growth is crucial for sustainable agriculture. Therefore, this study aimed to determine total and available Zn concentrations, to investigate the relationships between soil properties and Zn availability, and to characterize the spatial variability of Zn availability in the agricultural areas of Eskişehir and Kütahya provinces located in the Central Anatolia Region of Türkiye.

## Materials and methods

### Investigation region

**Fig. 1:**
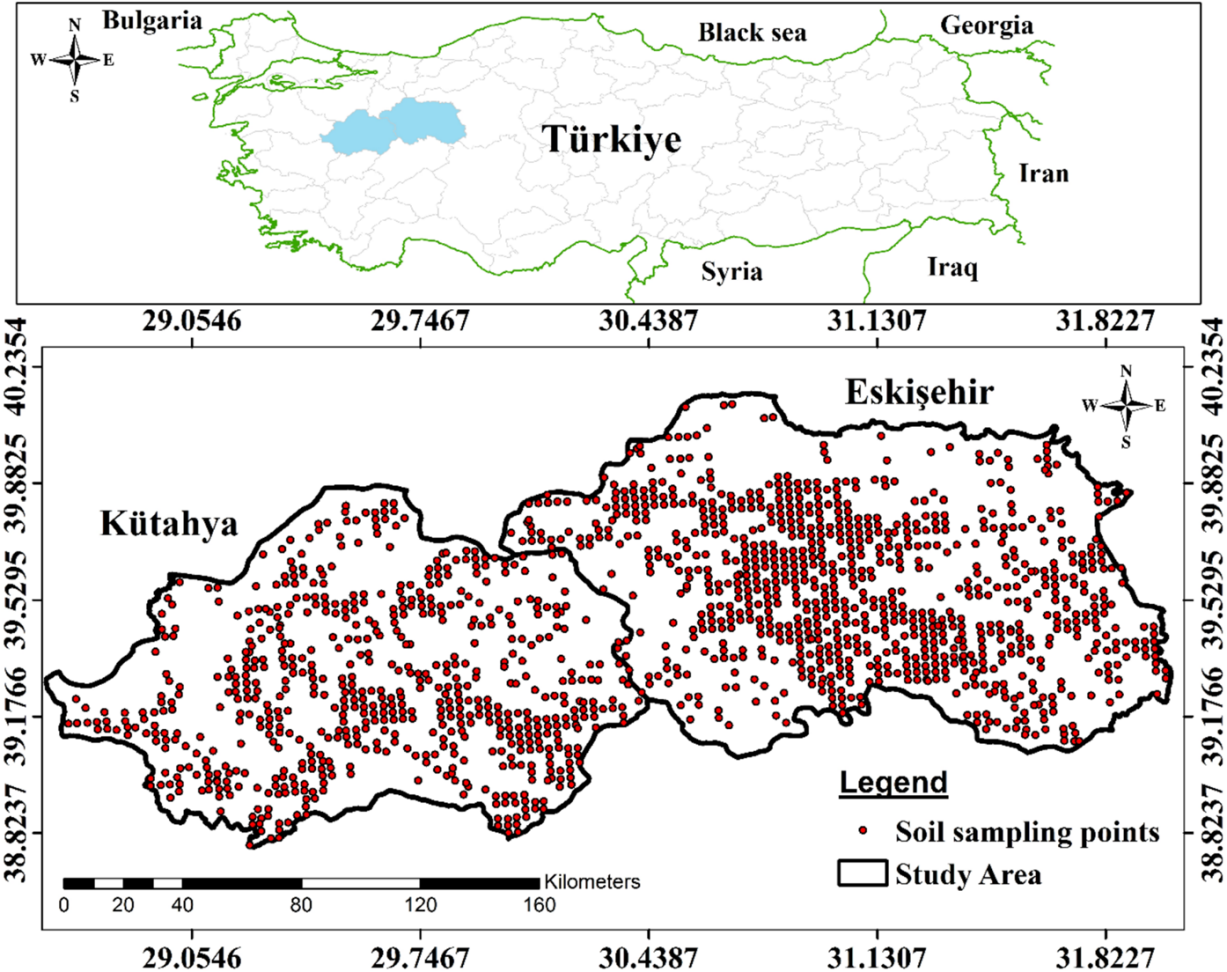
Location of the study area and distribution of soil sampling sites

This research was conducted in Eskişehir and Kütahya provinces of Türkiye (Fig. 1). Eskişehir is located in the northwest of Central Anatolia and covers an area of approximately 13,960 km^2^. Approximately 39% of Eskişehir’s land area is covered by agricultural activities. Eskişehir has typical continental climate characteristics with hot and dry summers and cold and snowy winters. The annual average temperature is 10.9 °C and the annual average total precipitation is 334 mm (Ağırbaş et al. 2023; Işınkaralar, 2024).

Kütahya, located in western Türkiye, has a surface area of 12,014 km^2^. Approximately 27% of the province’s total surface area consists of agricultural lands. Although Kütahya is located in the Mediterranean climate zone, it also has continental climate characteristics. Summers are hot and dry in the province, and winters are cold and rainy. The annual average temperature is 10.8 °C and the annual average total precipitation is 562 mm (Şahin et al. 2016).

### Soil sampling and analysis

Soil samples were collected from agricultural fields in Eskişehir and Kütahya provinces located in Central Anatolia between May and October of 2015 and 2016. A total of 1529 surface soil samples (0–30 cm) were collected from the study area (Fig. 1). Four random subsamples were collected at each sampling point and mixed thoroughly to form a composite soil sample. The collected soil samples were placed in nylon bags to be transported to the laboratory. In the laboratory, all samples were naturally air dried, stones and plant parts were removed, then ground and passed through a 2 mm sieve. The soil samples were analyzed for organic matter (OM), calcium carbonate (CaCO_3_), pH, available phosphorus (AP), available Zn (AZn), and total Zn (TZn). Many studies have shown that soil properties such as pH, CaCO_3_, AP and OM affect soil Zn concentration (Behera et al., 2011; Kabata-Pendias, 2010; Suganya et al., 2020), and therefore these parameters were analyzed in this study.

Soil OM concentration was determined by the wet oxidation method at 150 °C using a mixture of potassium dichromate and sulfuric acid (Walkley & Black, 1934). Soil pH was determined by measuring a mixture with a 1:2.5 soil/water ratio with a pH meter (Jenway, 3510). CaCO_3_ concentrations of the samples were measured using a Schibler calcimeter, while AP concentrations were determined using the molybdenum blue method (Olsen et al., 1954). TZn concentrations were measured using ICP-OES (Perkin Elmer Optima 8000, USA) after microwave digestion of the samples with a mixture of HCl and HNO_3_. Available Zn was extracted according to the DTPA method described by Lindsay and Norvell (1978) and its concentration was measured by ICP-OES. Zn availability ratio (Zn-AR) was calculated by the ratio of AZn to TZn concentration.

Reagents used in all analysis in this study were of analytical quality (Merck, Germany). Various quality control and assurance methods, including the use of blanks, replicates, calibration standards and a certified reference material (Loamy clay, CRM052), were utilized to ensure the quality of the analytical data. The average recovery rate of total Zn in the CRM was found to be 96%.

### Spatial analysis

In this study, spatial distribution maps of soil analysis results were created with the ArcGIS program (10.7), which is a geographic information system software. Different kriging methods (Ordinary kriging, simple kriging and universal kriging) were used to estimate the spatial distribution of soil analysis results. Kriging is one of the most accurate methods for generating soil characteristic estimation maps (Alaboz et al., 2021; Apriyono & Santoso, 2022). That is because of its high predictive power and its ability to minimize prediction error variation (Worsham et al., 2010). In current study, root mean square error (RMSE) was used to determine the most appropriate kriging method (Eq. 1).

1 $$\text{RMSE }=\sqrt{\frac{\sum {\text{(Zi-Z)}}^{2}}{\text{n}}}$$

In this formula; Zi is refer to the predicted value, Z is refer the observed value, and number of observations showed n.

### Statistical analysis

Kolmogorov- Smirnov normality test was employed to test the distribution of the data. Since the data did not show normal distribution, Spearman correlation analysis was chosen and a correlation plot was generated to show the relationships between the parameters. Violin plots were used to visualize the distribution of each parameter. OriginLab was used for plots and statistical analysis.

## Results and discussion

### Properties of soils

**Fig. 2:**
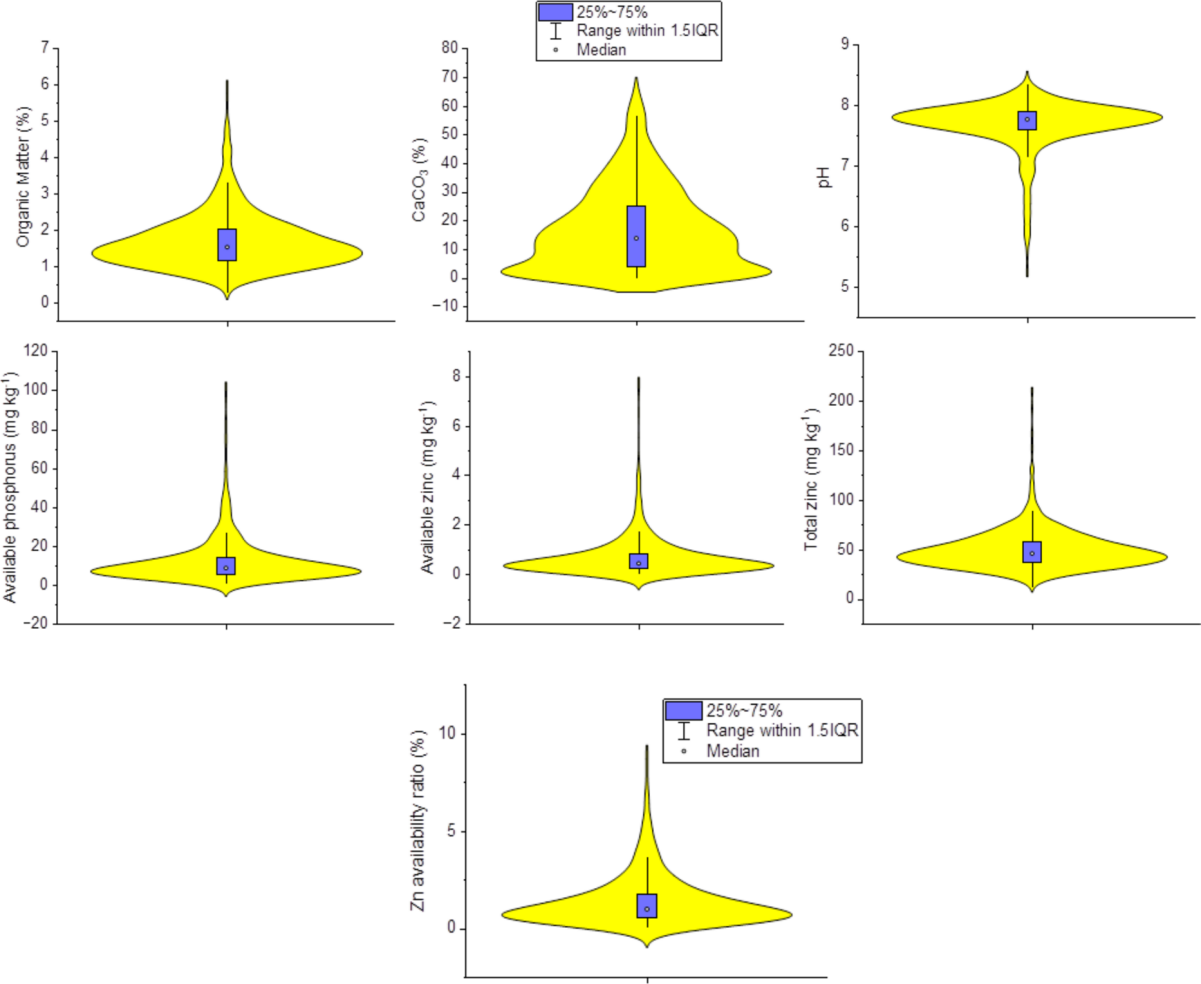
Violin plots of available Zn, total zinc and selected soil parameters investigated in this study

The basic statistic results of selected soil properties (CaCO_3_, pH, OM and AP), AZn and TZn concentrations of the soils in the study area are given in Table 1 and Fig. 2. It was determined that the coefficients of variation (CV) of AZn and Zn-AR exceeded 100%. However, the CV value of TZn was found to be 45.6%. This finding revealed that AZn concentration and Zn-AR had greater heterogeneity than TZn concentration in the study area. This may be because AZn and Zn-AR are more affected by extrinsic factors compared to TZn. Similar to the AZn and Zn-AR, the CV value of AP exceeded 100%. Previous studies reported that fertilizers, especially animal manure and phosphate-containing fertilizers, cause Zn and P accumulation in soils (Baltas et al., 2020; Marrugo-Negrete et al., 2017). Therefore, the high heterogeneity of TZn, AZn, AP and Zn-AR in the soils in the study area can be attributed to agricultural fertilizer use and soil parent material (Behera et al., 2011). Moreover, the CV values of OM and CaCO_3_ were found to be high (> 45%), indicating the high heterogeneity of the soils in the study area.

**Table 1:** Descriptive statistics for Zn concentrations and soil properties

	OM	CaCO_3_	pH	AP	AZn	TZn	Zn-AR
	(%)	(%)		(mg kg^−1^)	(mg kg^−1^)	(mg kg^−1^)	(%)
Minimum	0.26	0.00	4.77	1.07	0.01	11.74	0.03
Maximum	6.68	68.93	8.68	165.33	14.64	276.45	30.02
Mean	1.70	16.67	7.67	12.94	0.78	50.31	1.54
Standard deviation	0.79	14.65	0.45	14.71	1.22	23.03	2.01
Median	1.53	13.82	7.77	8.70	0.44	45.97	0.99
CV (%)	46.7	87.8	5.9	113.7	156.7	45.8	130.2

*OM* Organic matter, *AP* Available phosphorus, *AZn* Available zinc, *TZn* Total zinc, *Zn-AR* Available Zn ratio (AZn/TZn), *CV* Coefficient of variation

In this study, total Zn concentration ranged from 11.74 to 276.45 mg kg^−1^, with an average of 50.31 ± 23.03 mg kg^−1^. Based on the world soil average concentration of Zn (70 mg kg^−1^), it was found that 11.8% of the soil samples exceeded this concentration (Kabata-Pendias, 2010). Similarly, 13.1% of the samples were found to have concentrations above the European soil average (68.1 mg kg^−1^) (Kabata-Pendias, 2010) and 14.1% above the upper continental crust concentration (67 mg kg^−1^) (Rudnick & Gao, 2003). However, none of the samples exceeded the maximum allowable Zn concentration (300 mg kg^−1^) in soil (Kabata-Pendias, 2010). These findings reveal that Zn accumulation in the study area soils is low. It was determined that the AZn concentrations in the soils of the study area varied between 0.01 mg kg^−1^ and 14.64 mg kg^−1^. Previous studies define soils with DTPA-extractable Zn (AZn) concentration less than 0.5 mg kg^−1^ as Zn-deficient soils (Lindsay & Norvell, 1978). Considering this critical value, AZn deficiency was detected in 55% of the soil samples in the study area. This finding indicated that AZn concentration in the soils of the study area was low. That was an expected result since the study area is located in the semi-arid region. Moreno-Jiménez et al. (2022) reported that AZn concentrations decreased as aridity increased. Similarly, previous studies reported that there is a negative relationship between Zn concentration in agricultural soils and drought (Kabata-Pendias, 2010; Lindsay, 1979; Moreno-Jiménez et al., 2019). In addition, since drought reduces the amount of soil OM and increases soil pH, the amount of available microelements, including Zn, decreases (Moreno-Jiménez et al., 2019).

The availability of Zn in the soil is influenced by several factors, including parent material, soil texture, moisture, pH, OM, CaCO_3_ and phosphorus (Aytop et al., 2023; Suganya et al., 2020). One of the most important factors affecting the Zn concentration in soils is the Zn concentration in the parent material. In this respect, the low Zn concentration of soils in the study area is one of the important reasons for AZn deficiency (Suganya et al., 2020). Soil OM concentration is a vital indicator of soil health due to its beneficial effects on soil processes and properties. Soil OM is an important soil constituent which has an impact on crop yields as it improves and maintains soil health. Other physical, chemical and biological properties and processes are closely related to OM concentration (Lal, 2020). Previous studies have reported that available Zn tends to increase with increasing OM levels in soils (Moreno-Jiménez et al., 2019; Suganya et al., 2020). The threshold value indicating OM deficiency in agricultural soils has been reported as 2% (Ülgen & Yurtsever, 1995). In this study, the average OM concentration (1.7%) of the soils was found to be below the threshold value, indicating OM deficiency in the study area. In addition, OM deficiency was detected in 74.2% of the soil samples taken from the study area. Phosphorus (P) is a macronutrient that plays important roles in plant growth and participates in many metabolic reactions (Billah et al., 2019). Phosphorus deficiency in soils is one of the main limiting factors of plant growth in agricultural lands. Therefore, P fertilizers should be used regularly in agricultural soils to increase plant growth (Ateş et al., 2022). The AP concentrations in the study area ranged from 1.07 to 165.33 mg kg^−1^. Although the average AP concentration (12.94 mg kg^−1^) was above the 6 mg/kg threshold value for AP deficiency (Ülgen & Yurtsever, 1995), AP deficiency was detected in 29% of the soil samples. Soils containing more than 15% CaCO_3_ are considered calcareous (Bolan et al., 2023). In addition, calcareous soils are frequently found in semi-arid and arid regions (Bolan et al., 2023). In this study, the average CaCO_3_ concentration (16.67%) exceeding 15% indicates that the soils in the study area are calcareous. This is because the soil parent material in the study area is rich in limestone (Bolan et al., 2023). In addition, since annual precipitation in semiarid regions is relatively low compared to other regions, the Ca concentration of soils in semiarid regions is high (Weil & Brady, 2016; Yazhou et al., 2021), which causes CaCO_3_ accumulation in soils (Tisdale et al., 1985). The average pH value of the soils in the study area was found to be 7.5. Consistent with our findings, previous studies reported that the pH of calcareous soils was above 7 due to the high CaCO_3_ concentration of the soils (Bolan et al., 2023). High pH and CaCO_3_ levels in soils significantly reduce zinc availability by forming insoluble compounds such as Zn(OH)_2_ and ZnCO_3_ that cannot be used by plants (Alnaimy et al., 2023; Bolan et al., 2023; Kadifeci et al., 2024; Takrattanasaran et al., 2013). As a result, Zn deficiency in soils, which is primarily attributed to the high carbonate concentration, elevated pH, and low organic matter levels, is one of the important agronomic problems (Tsai et al., 1999; Behroozi et al., 2021; Budianta et al., 2023).

### Relationships between soil parameters

**Fig. 3:**
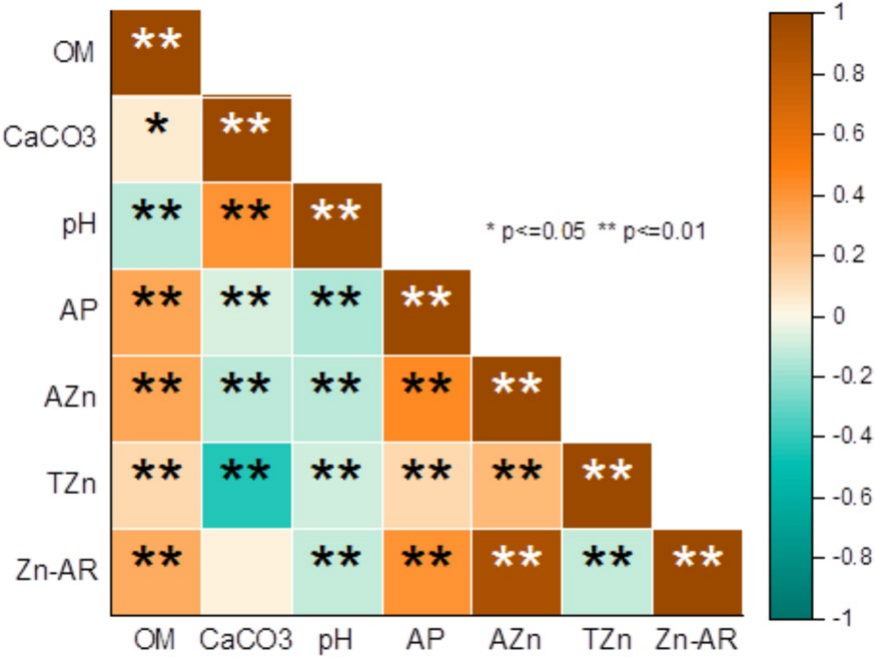
Correlation plot showing the relationships of available and total Zn with soil properties in the soils of the study area (n = 1529)

Spearman correlation analysis was conducted to characterize the relationships between TZn, AZn, Zn-AR and selected soil properties (OM, pH, CaCO_3_, and AP) (Fig. 3). As shown in correlation plot (Fig. 3), TZn was positively correlated with AZn, OM and AP (*p* < 0.01), while it was negatively correlated with pH, CaCO_3_ and Zn-AR (*p* < 0.01). Available Zn was negatively correlated with pH and CaCO_3_ (*p* < 0.01), while it was positively correlated with Zn-AR, OM and AP (*p* < 0.01). Similarly, Gupta et al. (2016) reported that there is a positive correlation between AZn and OM and TZn concentrations, while there is a negative correlation between AZn, CaCO_3_ and pH concentrations. However, Behera et al. (2011) found a positive correlation between AZn and pH, while Wei et al. (2006) found a positive correlation between AZn and CaCO_3_. In addition, AP was negatively correlated with pH and CaCO_3_ (*p* < 0.01), while it was positively correlated with OM and Zn-AR (*p* < 0.01). The strong positive correlation between AZn and Zn-AR (r = 0.917, *p* < 0.01) may be due to the interaction of other soil properties such as AP and OM because AZn had much higher positive correlation coefficients with OM and AP compared with TZn. The positive correlations between OM and TZn, AZn and AP indicate that the concentrations of TZn, AZn and AP increase with the increase of OM concentration. Similarly, Zamulina et al. (2021) found a positive correlation between TZn and OM. It has been reported that CaCO_3_ in calcareous soils limits the availability of Zn and P (Bolan et al., 2023). This may be a good explanation for the negative correlations between CaCO_3_ and AZn and AP. Similarly, Vasu et al. (2021) found a negative relationship with CaCO_3_ and AZn in their study on 1508 soil samples. Likewise, Shukla et al. (2016) determined that Zn was negatively correlated with CaCO_3_ and pH and positively correlated with OM. In addition, the findings obtained in our study are consistent with the findings of Katyal and Sharma (1991), who determined that soil pH, CaCO_3_ and OM have a strong effect on the distribution of micronutrients, and White and Zasoski (1999), who found that organic matter increases the amount of Zn in the soil by providing soluble chelating agents and reducing the oxidation of cations.

### Spatial distribution

To construct spatial distribution maps of soil parameters investigated in this study (TZn, AZn, AP, Zn-AR (AZn/TZn), CaCO_3_, OM and pH), ten different kriging models were used and the RMSE values of these models were determined (Table 2). It was found that Simple Kriging was the most suitable method for all soil parameters except pH. Many researchers reported that the Simple Kriging method showed the least variance in estimating the values of unsampled areas (Alaboz et al., 2021; Motaghian & Mohammadi, 2011).

**Table 2:** Cross-validation and their RMSE values according to Kriging

	Ordinary			Simple			Universal		
	Gau	Exp	Sph	Gau	Exp	Sph	Gau	Exp	Sph
AZn	1.093	1,092	1.095	**0.903**	0.932	0.900	1.093	1.092	1.095
TZn	0.982	1.051	1.053	**0.874**	0.936	0.883	0.982	1.051	1.053
Zn-AR	1.182	1.199	1.221	1.007	1.033	**1.003**	1.182	1.199	1.221
AP	1.012	1.039	1.039	**0.992**	1.057	1.012	1.012	1.039	1.039
CaCO_3_	0.985	0.986	0.984	**0.912**	0.957	0.918	0.985	0.986	0.984
OM	0.783	0.781	0.782	0.772	**0.771**	0.772	0.784	0.784	0.784
pH	0.377	0.369	0.371	0.379	0.373	0.377	0.377	**0.369**	0.371

*Gau.* Gaussian, *Exp*. Exponential, *Sph*. Spherical

**Fig. 4:**
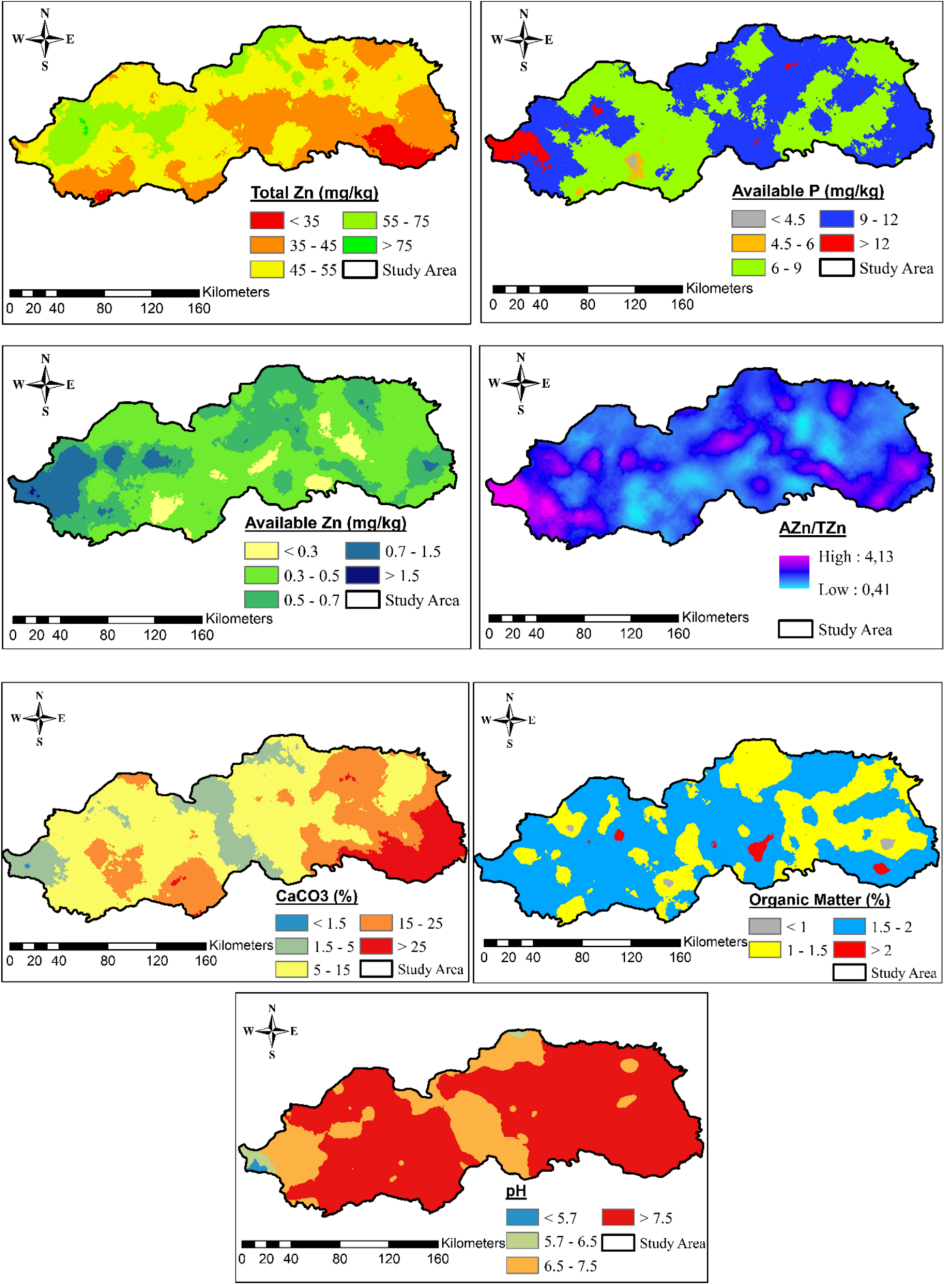
Spatial distribution maps of TZn, AP, AZn and Zn-AR (AZn/TZn), CaCO_3_,OM and pH in the study area

When the spatial distribution maps of TZn and AZn concentrations were examined, some differences in their distribution trends were observed. Total Zn concentrations were found to be higher in the northern and northwestern regions and lower in the southeastern region of the study area (Fig. 4). Available Zn showed higher concentrations in the western region and lower concentrations in the southern and central regions (Fig. 4). Similar to AZn, Zn-AR showed higher values in the western region and lower values in the central region of the study area. Also, as with AZn and Zn-AR, AP showed higher concentrations in the western region (Fig. 4). This indicated that Zn-AR was influenced by both AZn concentration and other soil properties such as AP. However, CaCO_3_ concentrations exhibited opposite distribution trends to TZn, showing lower concentrations in the western region and higher values in the southeastern region of the study area.

## Conclusions

The study area exhibited the greatest variability in the parameters of AZn, Zn-AR, and AP. The level of zinc contamination was assessed as low when compared to the upper continental crust and the world average concentrations of Zn, with none of the soil samples surpassing the maximum allowable Zn level. A deficiency in available zinc was identified in 55% of the soil samples. Two critical soil characteristics that influence the variability of AZn and Zn-AR were found to be available phosphorus and organic matter. Higher total zinc concentrations were recorded in the northern and northwestern regions. The distribution trends for available zinc and Zn-AR were alike, showing elevated values in the western region. This research offers insights into identifying and mapping the prevalence of zinc deficiency in areas with intensive cultivation. Specifically, the distribution maps created for zinc and organic matter serve as a primary resource for developing regionally tailored micronutrient management strategies and future soil sampling plans in the semi-arid Central Anatolian Region. Future investigations could involve collecting plant samples alongside soil samples, providing crucial information to decision-makers regarding the transfer of microelements from soil to plants, and facilitating the creation of new strategies for plant nutrition.

## Data Availability

No datasets were generated or analysed during the current study.

## Declarations

### Permissions

All figures in this manuscript are original figures produced by the authors and have not been published elsewhere.
